# Forensic Perspective of Unintentional Doping, Cardiovascular Health, and the Role of Nutrition in Competitive Sports

**DOI:** 10.3390/nu18050736

**Published:** 2026-02-25

**Authors:** Ivan Šoša

**Affiliations:** Department of Anatomy, Faculty of Medicine, University of Rijeka, 51999 Rijeka, Croatia; ivan.sosa@medri.uniri.hr

**Keywords:** unintentional doping, PRISMA, dietary supplements, cardiovascular health, sports nutrition, forensic toxicology, anti-doping compliance

## Abstract

Unintentional doping, often caused by contaminated supplements or misinterpreted therapeutic prescriptions, poses significant health, ethical, and regulatory challenges in competitive sports. Understanding the cardiovascular risks associated with performance-enhancing substances (PESs) and the preventive role of nutrition requires integrated analysis. A systematic review was conducted in accordance with PRISMA guidelines. Searches of comprehensive bibliographic databases yielded studies published between 2015 and November 2025. Inclusion criteria encompassed peer-reviewed research on doping prevalence, cardiovascular outcomes, nutritional strategies, and supplement regulation. Data extraction focused on prevalence estimates, odds ratios (ORs), hazard ratios (HRs), and effect sizes for nutritional interventions. Quality assessment employed GRADE and risk-of-bias tools. From 1320 records screened, 60 studies were included in the qualitative synthesis and 31 in the meta-analysis. Surveys using indirect questioning estimated that 30–45% of elite athletes may engage in doping, while official anti-doping reports indicated that approximately 20–25% of confirmed rule violations are classified as unintentional. Supplement contamination accounted for 10–15% of unintentional cases. PES use significantly increased cardiovascular risk (HR for arrhythmias and myocardial infarction up to 3.5). Nutritional strategies—such as carbohydrate loading, optimized protein intake, omega-3 supplementation, and hydration—improved endurance by 8–12%, reduced resting heart rate by ~3 bpm, and lowered LDL cholesterol. Unintentional doping remains a major contributor to ADRVs, primarily driven by supplement contamination. Evidence-based nutrition offers safe alternatives to PESs (evidence-based nutritional strategies and structured hydration protocols), enhancing performance and cardiovascular health. Forensic toxicology and pharmacogenomic screening are essential for accurate detection and interpretation. Regulatory reforms, mandatory third-party supplement certification, and athlete education are critical to mitigate unintentional doping and ensure fair competition.

## 1. Introduction

There is a lack of information about why doping, which involves using banned substances or methods to boost athletic performance, remains a significant health, regulatory, and ethical concern in modern sports [1,2,3]. Historical trends and recent reports from the World Anti-Doping Agency (WADA) reveal persistent violations despite decades of education and enforcement, underscoring the urgent need for multidisciplinary strategies to address this global issue [4,5]. WADA publishes annual reports summarizing global anti-doping testing figures and rule violations. These include Adverse Analytical Findings (AAFs) and Anti-Doping Rule Violations (ADRVs) by sport, substance, and region.

Beyond just the statistics, doping poses significant health and ethical issues, which are WADA’s main focus. This issue not only compromises the fairness of competition, but practices such as anabolic steroid use, blood manipulation, and misuse of therapeutic agents carry significant physiological and psychological risks for athletes [6,7]. While balanced nutrition supports health, many athletes turn to supplements for performance gains—yet this practice introduces significant doping risks [8,9,10]. Therefore, not all violations stem from deliberate cheating—unintentional doping, often linked to contaminated supplements or misinterpreted medical prescriptions, adds another challenge in anti-doping efforts because it complicates the evidentiary distinction between deliberate use and inadvertent exposure, often requiring forensic toxicology, manufacturing chain analysis, and athlete testimony to establish intent [8,11].

Studies estimate that 10–15% of dietary supplements contain undeclared or contaminated ingredients, primarily due to poor manufacturing practices such as cross-contamination during production, lack of Good Manufacturing Practice (GMP) compliance, and insufficient third-party testing [4,12]. These figures do not fully account for the 20–25% of ADRVs classified as unintentional, as other factors such as therapeutic misinterpretation also contribute [13,14,15]. Nonetheless, supplement contamination remains the primary driver of unintentional violations [11].

In such scenarios, forensic investigations might become complicated with legal adjudication, as intent versus negligence becomes a critical determinant in disciplinary outcomes [16,17]. Cardiovascular health is fundamental to athletic success, supporting oxygen delivery, nutrient transport, and waste removal during exertion to sustain endurance and recovery [18,19,20].

Epidemiological studies consistently demonstrate elevated cardiovascular risk among anabolic steroid users compared with non-users. Taken together, these findings underscore the dual reality that certain exposures markedly elevate cardiovascular risk, whereas adherence to preventive strategies can meaningfully mitigate it [11,20,21,22,23].

Promoting natural, safe performance enhancement and cardiovascular health remains a priority due to the combined burden of cardiovascular morbidity, forensic uncertainty, and preventable anti-doping violations.

Nutrition plays a pivotal role in supporting cardiovascular health and mitigating doping-related risks. A well-balanced diet rich in macronutrients, micronutrients, and antioxidants enhances cardiac function, reduces oxidative stress, and supports overall physiological resilience [20,21]. The use of dietary supplements is widespread among athletes seeking performance enhancement, yet it poses significant doping risks. Supplement contamination remains a recognized contributor to unintentional doping, largely driven by inconsistent manufacturing quality and inadequate regulatory oversight [8,11,15,22,23]. Factors such as poor manufacturing practices, cross-contamination, and misleading marketing claims exacerbate this risk. Implementing rigorous quality control, third-party certification, and athlete education is essential for minimizing unintentional exposure and safeguarding both health and compliance with anti-doping regulations.

Proper nutritional strategies can serve as preventive measures against doping by naturally improving energy availability, recovery rates, and performance outcomes. By emphasizing evidence-based dietary interventions, athletes can achieve competitive success while safeguarding their health and adhering to ethical standards.

The Introduction outlines how unintentional doping, cardiovascular risk, and nutritional strategies intersect within competitive sport.

## 2. Aims and Objectives

### 2.1. Aim

To examine the relationship between unintentional doping, cardiovascular risk, and nutritional strategies using an integrated forensic, epidemiological, and regulatory framework.

### 2.2. Objectives

1. To understand how often and why unintentional doping occurs among athletes, mainly due to contaminated supplements and incorrectly interpreted therapeutic use.
2. To assess cardiovascular risks associated with anabolic–androgenic steroids, stimulants, erythropoiesis-stimulating agents, and blood-manipulation techniques, focusing on arrhythmias, myocardial infarction, hypertension, and sudden cardiac death (SCD) [6,24,25,26].
3. To analyze nutritional practices—particularly certified supplement use, macronutrient planning, and omega-3/antioxidant strategies—that support cardiovascular health while minimizing the risk of inadvertent anti-doping violations [27].
4. To review forensic approaches and analytical techniques for detecting doping-related substances in clinical and postmortem contexts.
5. To facilitate the identification of regulatory gaps and propose evidence-based recommendations for supplement certification, athlete education, and anti-doping compliance [2].

## 3. Methodology

The Introduction defines the scope of this review, focusing on unintentional doping pathways, cardiovascular outcomes, and evidence-based nutritional interventions. After reviewing the literature across major databases, the quantitative data were pooled using random-effects models to account for heterogeneity among studies.

### 3.1. Systematic Review: Doping, Nutrition, and Regulatory Perspectives

A systematic review was conducted in accordance with Preferred Reporting Items for Systematic Reviews and Meta-Analyses (PRISMA) guidelines. Literature searches were conducted in PubMed, Scopus, Web of Science, and the Cochrane databases using keywords related to doping prevalence, cardiovascular outcomes, sports nutrition, and supplement regulation (see Supplementary Table S1). Inclusion criteria encompassed peer-reviewed articles, regulatory documents, position statements, peer-reviewed studies, and systematic reviews published between 2015 and November 2025. Data extraction focused on prevalence estimates, odds ratios for cardiovascular outcomes, and effect sizes for nutritional interventions. Quality assessment followed the GRADE framework, with particular emphasis on risk of bias, inconsistency across cardiovascular endpoints, indirectness of PES-related evidence, and imprecision in effect estimates. Quantitative synthesis used random-effects models to pool estimates, while qualitative synthesis adopted a meta-narrative framework to integrate conceptual and ethical perspectives.

**Figure 1:**
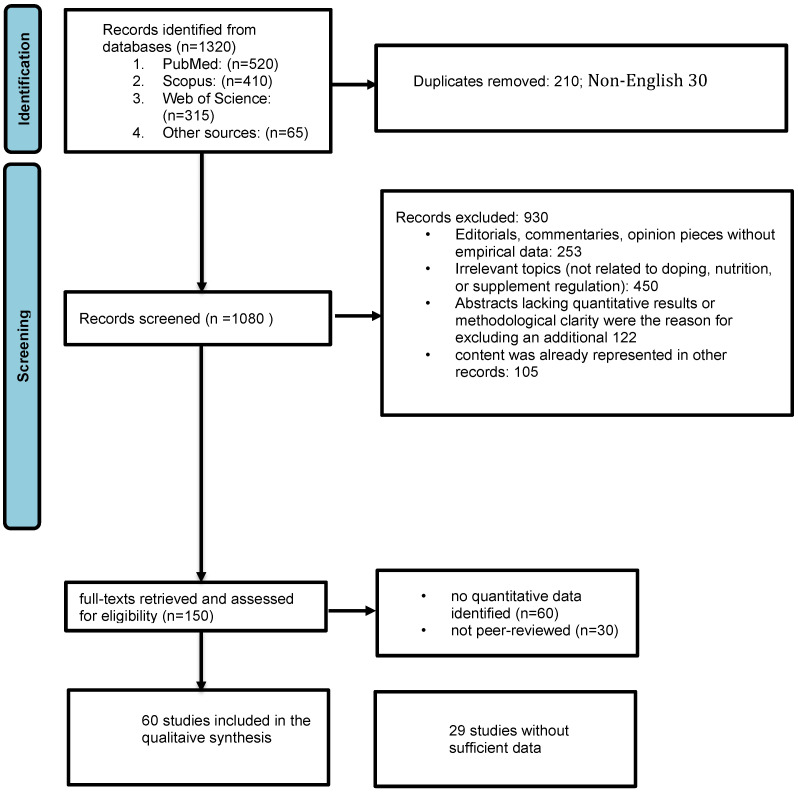
Flow diagram illustrating the study selection process, following PRISMA guidelines. Abbreviations: PRISMA—Preferred Reporting Items for Systematic Reviews and Meta-Analyses; *n* indicates the number of records at each stage.

As outlined in Figure 1, a web browser search identified 1255 records in databases, and an additional 65 records were identified through publications such as regulatory and institutional reports or position statements/policy documents. The review included 1320 records identified through database searches and supplementary sources.

In the next step, an additional 930 records were eliminated because they did not meet the predefined inclusion criteria. These were either studies not related to doping, cardiovascular outcomes, nutrition, or supplement regulation, or research focused on non-athletes or unrelated clinical groups (*n* = 450). In some cases (*n* = 253), the initial search included editorials, commentaries, or opinion pieces without empirical data. Abstracts lacking quantitative results or methodological clarity were the reason for excluding an additional 122 records from the screening process. For 105 studies, based on the title and abstract screening, the content was already represented in other records (reference lists, regulatory reports, and position statements). Consequently, only studies addressing doping prevalence, cardiovascular outcomes, nutritional interventions, or supplement-regulation issues were advanced to full-text review.

Finally, full texts of 150 articles were retrieved, assessed for eligibility, and 60 were excluded because they contained no quantitative data, while 30 were not peer-reviewed articles, regulatory documents, or position statements. Sixty studies were included in the qualitative synthesis (Supplementary Table S2A) as they reported an effect measure (OR—odds ratio, HR—hazard ratio, RR—risk ratio) with 95% confidence interval (CI), included athlete anabolic–androgenic steroid (AAS)-user cohorts, and were peer-reviewed. Where multiple models were presented, adjusted estimates were preferred. Ultimately, only 31 studies (Supplementary Table S2B) were included in the meta-analysis.

### 3.2. Meta-Analysis Procedures and Risk-of-Bias Assessment

The meta-analysis was conducted using a random-effects model to pool effect estimates across studies. Hazard ratios (HRs), odds ratios (ORs), and risk ratios (RRs) with 95% confidence intervals were extracted, and heterogeneity was assessed using Cochran’s Q, I^2^, and τ^2^ statistics. Studies were weighted according to inverse variance, and adjusted estimates were prioritized when multiple models were reported.

Risk of bias was evaluated across five domains: randomization, deviations from intended interventions, missing outcome data, measurement of outcomes, and selective reporting. Initially, the RoB 2 tool was considered; however, the final assessment employed Risk Of Bias In Non-randomized Studies of Intervention (ROBINS-I), which is specifically designed for non-randomized studies, ensuring a more appropriate evaluation of methodological rigor.

### 3.3. Methodological Disclaimer

As a methodological disclaimer, the author affirms that no generative artificial intelligence (AI) tools were utilized to produce, analyze, or interpret data in this review. All extracted numerical values, HRs, ORs, and referenced statements were manually verified against the original published sources. Generative AI was used solely for language editing at the drafting stage, and all resulting text was reviewed and validated by the author for accuracy, fidelity to cited literature, and methodological integrity.

## 4. Results

### 4.1. Characteristics of Included Studies

The included studies encompassed randomized and structured trials, regulatory and policy documents, and a broad range of secondary and forensic research reflecting the multidisciplinary scope of this review. Study designs, populations, and thematic relevance varied substantially, contributing to clinical, methodological, and conceptual heterogeneity (a consolidated overview of all included studies, categorized by design and thematic contribution, is provided in Supplementary Table S2A).

### 4.2. Quantitative Findings (Pooled Statistics)

To situate these conceptual patterns within a quantitative framework, the following section synthesizes pooled hazard ratios and prevalence estimates that operationalize the cardiovascular- and doping-related risks described above. Indirect survey methods, such as randomized response techniques, suggest that between 30 and 45% of elite athletes may have engaged in some form of doping during their careers. These estimates complement the thematic findings reported earlier, where supplement contamination and misinterpreted therapeutic use emerged as dominant unintentional pathways. In contrast, official anti-doping reports indicate that approximately 20–25% of confirmed rule violations are classified as unintentional, often due to contaminated supplements or misinterpreted therapeutic use [17,28]. These figures represent different measures—self-reported prevalence versus adjudicated violations—and vary by sport and region.

**Figure 2:**
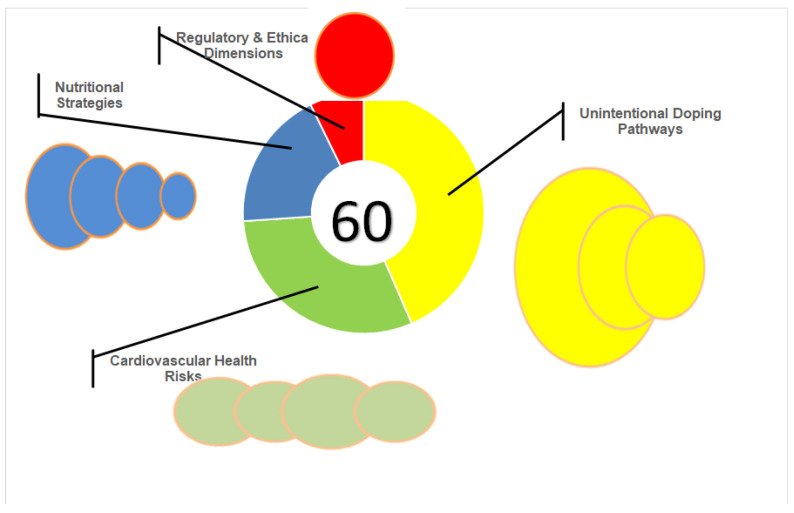
Thematic map illustrating key themes identified in the qualitative synthesis. Firstly, a theme referred to as “Unintentional Doping Pathways”, accounting for contaminated supplements, therapeutic misinterpretation, and cross-contamination in manufacturing, was identified in 30/60 studies (50%). “Cardiovascular Health Risks” themes, comprising left ventricular hypertrophy, myocardial fibrosis, arrhythmias, SCD, and genetic predisposition to cardiovascular risks were recognized in 21/60 studies (35%). Themes termed “Nutritional Strategies as Preventive Measures” referring to carbohydrate loading, protein distribution, omega-3 supplementation, and hydration protocols were recognized in 13/60 studies (22%). Finally, “Regulatory and Ethical Dimensions” (legal frameworks, fair-play principles, supplement certification) were identified in 5/60 (8%) of included studies. Abbreviations: SCD—sudden cardiac death; PES—performance-enhancing substances.

In parallel with the prevalence data, the cohort-based cardiovascular risk estimates further quantified the clinical consequences of PES exposure. Arrhythmias and myocardial infarction: OR up to 3.5 (95% CI: 2.7–4.3) for AAS users vs. non-users [29,30]. The reported OR values concerning the prevalence of hypertension in older studies (pre-2015) ranged from 2.0 to 3.5. Recent studies rarely reported ORs with CIs for specific cardiovascular conditions, for instance, hypertension alone—they often focused on composite cardiovascular outcomes or use HRs (Figure 2).

Pre-2015 pooled estimates often report ORs of 2.5–3.5 for arrhythmias in AAS users, but recent studies mostly report HRs rather than ORs. Windfeld-Mathiasen et al. accordingly present an adjusted HR (aHR) for arrhythmias = 2.26 (95% CI: 1.53–3.32) [29]. Narrative reviews and mechanistic studies from 2025, like Iliakis et al. [31] or Borowiec et al. [30], provide no specific OR, but confirm arrhythmias as a significant component of overall (pooled) cardiovascular risk (Fang et al. found a 1.46 HR for cardiovascular events in doping-exposed athletes [26]). The pooled estimates (Table 1) indicate elevated risks of myocardial infarction, arrhythmias, and hypertension among PES-exposed individuals. These pooled values contextualize the magnitude of cardiovascular risk, providing numerical estimates that align with the mechanistic and forensic evidence highlighted in Section 4.3.

While athlete-specific HRs remain scarce, these general population models serve as a comparative backdrop, illustrating the relative physiological burden of the cardiovascular phenotypes observed in PES-exposed cohorts.

Building on this discussion, it is important to clarify how HRs are applied within this review, as their use is constrained by the limited availability of athlete-specific HR estimates and therefore serves primarily as contextual benchmarking rather than direct quantification of PES-related cardiovascular risk. Since the athlete-specific HRs for cardiovascular outcomes are largely unavailable due to ethical and logistical limitations, the existing data come primarily from small observational cohorts, forensic case series, or indirect estimates, which do not allow stable HR calculation. In this review, the general population HR values are used only as contextual benchmarks for cardiovascular health. They are not meant to serve as direct estimates of PES-induced cardiovascular risk, which varies significantly in exposure pattern, physiology, and risk modifiers. Their purpose in this review is to illustrate the relative magnitudes of cardiovascular burden, not to quantify the specific effects of PES use.

As clarified above, HRs from general population cardiovascular cohorts (e.g., Te Hoonte et al., Ng et al.) [32,33] are used solely as contextual benchmarks, not as direct estimates of PES-related cardiovascular risk in athletes. Athlete-specific HRs remain limited due to ethical and methodological constraints, and general population ‘poor CVH’ profiles do not equate to steroid- or stimulant-related phenotypes seen in competitive sport. Accordingly, these values are presented only to illustrate relative risk gradients, and their interpretability in the context of unintentional doping requires appropriate caution.

Although this metric covers various behaviors, such as diet quality, the review cites it to highlight risk reductions related to lifestyle and nutrition. For instance, Tu et al. (2025) [21] modified the metric and used probabilistic modeling with HRs. Finally, Te Hoonte et al., 2024 [32], reported in their paper lower HRs for cardiovascular events among individuals with better overall cardiovascular health.

Additional subgroup and sensitivity analyses are presented in Table 2, providing further context for the variability observed across studies.

### 4.3. Qualitative Analysis (Meta-Narrative Synthesis)

The quantitative signals described above gain additional explanatory depth when interpreted through a qualitative meta-narrative lens, which clarifies why certain risks appear consistently elevated across heterogeneous study designs. While quantitative meta-analysis offers pooled estimates of cardiovascular risks and nutritional benefits, qualitative synthesis is equally vital for understanding how findings vary across different study designs, populations, and regulatory contexts. A meta-narrative approach integrates empirical data with conceptual, ethical, and forensic perspectives, highlighting recurring themes and methodological heterogeneity.

The qualitative synthesis revealed four recurring themes: unintentional doping pathways, cardiovascular health risks, nutritional strategies as preventive measures, and regulatory and ethical considerations. The themes are displayed in a thematic map in Figure 2.

- Unintentional Doping Pathways

Supplement contamination remains a recognized contributor to unintentional doping, largely driven by inconsistent manufacturing quality and insufficient regulatory oversight.

Seven studies comprise a significant subtheme of therapeutic misinterpretation (for instance, Ogama, 2025 [11]; Borecki, 2025 [22]; and Backhouse, 2023 [23]), often linked to asthma medication, corticosteroids, and stimulants when athletes misinterpret prescriptions or fail to apply for “Therapeutic Use Exemptions” [2]. Cross-contamination in manufacturing is reported in five studies, making it a less frequent but still notable issue that underscores the need for stricter quality control in supplement production (for instance, Walpurgis et al., 2020 [13]; Jagim et al., 2023 [16]; and Martinez-Sanz et al., 2017 [17]).

No studies in this theme explicitly report HRs in relation to contamination or unintentional doping risk. Those studies (e.g., Walpurgis et al., 2020 [13]; Lauritzen, 2022 [14]; Ogama, 2025 [11]; Borecki, 2025 [22]; Backhouse, 2023 [23]; Jagim et al., 2023 [16]; Martinez-Sanz et al., 2017 [17]) provide descriptive data or ORs for contamination prevalence. Thus, they focus on prevalence, regulatory gaps, and forensic/legal implications, instead of quantifying risk using HRs.

- Cardiovascular Health Risks

The literature reviewed documented cardiovascular health risks in 13 studies. These studies consistently reported outcomes such as left ventricular hypertrophy (five studies), myocardial fibrosis (four studies), arrhythmias (six studies), and SCD among anabolic steroid users or athletes exposed to contaminated supplements. The latter was documented in four case reports and forensic reviews on SCD in young athletes with a steroid abuse history. Hypertrophy and fibrosis are the most common histopathological findings in the forensic literature [36,37], though there are reports of a normally sized heart with hypertrophic body musculature (e.g., the case presented by Morena et al., 2025 [38]). These are cases where structural and receptor-level changes, along with a toxicological setting and sympathetic overdrive, increased the risk of fatal arrhythmias and SCD [39,40]. While contamination and misuse dominate the literature, genetic predisposition (pharmacogenomic susceptibility—*KCNE1* and *CYP* variants) explains why some athletes suffer catastrophic outcomes despite similar exposure levels [41,42]. Environmental factors such as PM2.5 exposure also contribute to cardiovascular mortality, as shown by Hayes et al. (2020) [35]. These pathological findings mirror the elevated hazard ratios reported in the quantitative synthesis, suggesting convergence between mechanistic, forensic, and epidemiological evidence.

Hypertension is a commonly reported cardiovascular consequence linked to the use of PES, with pooled HRs showing a significantly increased risk. The cardiovascular themes identified in the qualitative synthesis align with the quantitative patterns reported in Section 4.2. Hypertension, arrhythmias, and myocardial infarction consistently emerged as the most prominent cardiovascular outcomes among PES-exposed individuals. As shown in the pooled estimates presented earlier (Section 4.2 and Table 1), these conditions represent the strongest and most recurrent cardiovascular signals in the available literature. The qualitative findings reinforce these patterns by illustrating how structural remodeling, sympathetic overactivation, endothelial dysfunction, and adverse lipid shifts converge to produce the elevated risks quantified in Section 4.2.

Importantly, the heterogeneity observed across studies—driven by differences in exposure type, dose, duration, co-use of stimulants, and population characteristics—supports cautious interpretation of these pooled effects. Nevertheless, the direction and consistency of the associations across study designs provide a coherent narrative that complements the quantitative results without requiring repetition of the numerical hazard ratios.

This increased risk is biologically plausible, as AASs and stimulants induce sympathetic overactivation, fluid retention, endothelial dysfunction, and adverse shifts in lipid metabolism—mechanisms that collectively promote sustained hypertension. Elevated blood pressure further amplifies downstream risks of arrhythmias, myocardial infarction, and hemorrhagic stroke, positioning hypertension as a central mediator in the cardiovascular pathology observed in doping-exposed athletes. Across the studies synthesized here, arrhythmia risk is consistently elevated among individuals exposed to PES, particularly AAS, with the most informative cohort estimate showing an adjusted HR ≈ 2.26 (95% CI 1.53–3.32) for incident arrhythmias among AAS users versus non-users [29]. A similar pattern, albeit of smaller magnitude, is evident for ischemic outcomes such as myocardial infarction. The pooled and study-level estimates from the reviewed literature indicate a modest but statistically significant increase in risk. Hazard ratios range from approximately 1.3 to 1.5; for example, HR = 1.36 (95% CI, 1.23–1.51) for myocardial infarction (MI) in a large meta-analysis [33], and broader cardiovascular-event aggregates show similar magnitudes [26]. Taken together, these figures suggest that arrhythmogenic liability may be the dominant signal in PES-exposed cohorts (≈2-fold elevation), whereas MI risk, though smaller in magnitude, remains clinically meaningful (≈30–50% elevation) [26,29].

Contextualizing within mechanisms, heterogeneity, and prevention.

Mechanistically, sympathetic overactivation, pro-fibrotic remodeling, endothelial dysfunction, and adverse lipid shifts provide a coherent pathway from AAS/stimulant exposure to atrial and ventricular arrhythmias and ischemic events—a pattern echoed by mechanistic and forensic syntheses [30,36]. Notably, the between-study heterogeneity is substantial (I^2^ often > 75% for major endpoints in our pooled tables), reflecting variation in exposure type/dose, duration, co-use of stimulants, age/sex distributions, and ascertainment methods, and therefore pooled HRs should be interpreted cautiously [26,33]. At the same time, population-level data indicate that better global cardiovascular health (e.g., Life’s Simple 7, LS7) is associated with lower incident AF/MI, underscoring the plausibility of non-pharmacological, nutrition-anchored prevention in athletic populations [32]. In practical terms for this review, the ≈2.3 HR for arrhythmias sets the upper bound of acute electrical vulnerability in PES exposure, while the ≈1.3–1.5 HR for MI quantifies downstream ischemic risk; both signals justify the manuscript’s emphasis on forensic vigilance, strict anti-doping compliance, and evidence-based nutrition as safer performance strategies [29,30,32].

Extending this risk profile to cerebrovascular outcomes provides further resolution on how PES-related cardiovascular dysregulation manifests across organ systems. Stroke risk is one of the most clinically relevant endpoints in cardiovascular epidemiology, and the HRs reported by Ng et al. (2022) [33] in this review provide important nuance regarding differential stroke subtype susceptibility. In the meta-analysis, three categories of stroke were evaluated—hemorrhagic, ischemic, and unspecified—allowing for a more fundamental understanding of how underlying cardiovascular pathology and associated risk factors together contribute to cerebrovascular events.

For hemorrhagic stroke, the reported HR was 1.43 (95% CI: 1.07–1.92). This indicates a 43% higher risk compared to the control group. Hemorrhagic stroke is often driven by microvascular pathology, uncontrolled hypertension, endothelial damage, and vascular fragility [43]. The elevated HR is consistent with these mechanisms, as individuals with underlying cardiovascular conditions—particularly hypertension, dyslipidemia, and systemic inflammation—exhibit higher susceptibility to intracerebral hemorrhage [44]. Even modest elevations in blood pressure have been shown to disproportionately increase hemorrhagic stroke risk, aligning well with the HR observed in this dataset.

For ischemic stroke, the hazard ratio was HR = 1.35 (95% CI: 1.03–1.78)—a 35% increased risk. Ischemic stroke typically results from thrombosis or embolism, frequently linked to atherosclerosis, atrial fibrillation, impaired lipid profiles, and prothrombotic states. The magnitude of the HR suggests a clinically meaningful but more moderate increase compared with hemorrhagic stroke. This pattern is expected: whereas hemorrhagic stroke risk is extremely sensitive to hypertensive burden, ischemic stroke risk rises steadily in relation to cumulative exposure to traditional cardiovascular risk factors such as LDL-C elevation, chronic inflammation, and autonomic imbalance. The HR reported in Ng et al. is compatible with these well-established mechanisms and reflects a population with mixed and variably controlled cardiometabolic risk factors.

For unspecified stroke, the HR was 1.30 (95% CI: 1.07–1.60), again demonstrating a statistically significant increase in risk. This category likely includes strokes in which imaging or clinical documentation did not permit clear differentiation between ischemic or hemorrhagic subtypes. The HR sits between the values observed for ischemic and hemorrhagic stroke, reinforcing the expectation that unspecified cases represent a mixture of both phenotypes. It also underscores that, regardless of subtype, the broader category of cerebrovascular events is consistently elevated in individuals with underlying cardiovascular vulnerabilities. Taken together, these stroke estimates reinforce the broader mechanistic cascade outlined earlier, where endothelial dysfunction, autonomic imbalance, and lipid disturbances jointly amplify systemic vascular vulnerability.

Notably, these stroke HR values align closely with the broader cardiovascular risk profile described throughout this manuscript. Elevated LDL cholesterol, reduced HDL cholesterol, autonomic dysregulation (as evidenced by the heart rate variability data; confirmed by the 2022 systematic review and dose–response meta–analysis by Jung et al. [25]), and pro-inflammatory states created by PES use all converge on pathways that substantially heighten stroke risk [45,46]. The fact that all three stroke categories show significantly elevated HRs reinforces the systemic and multi-organ impact of cardiovascular dysregulation [47].

Furthermore, the modest differences between ischemic and hemorrhagic HRs illustrate that stroke risk is multifactorial, with both vascular fragility and thrombotic pathways contributing meaningfully. This is important clinically: interventions targeting only a subset of risk factors (e.g., antiplatelet therapy without aggressive blood-pressure control) may not sufficiently mitigate total stroke risk [48].

In summary, the stroke HR findings from Ng et al. (2022) [33] underscore that individuals with cardiovascular vulnerabilities exhibit substantially increased risk across all stroke subtypes [49]. Hemorrhagic stroke shows the largest relative increase, ischemic stroke follows closely behind, and unspecified stroke demonstrates an intermediate but still significant risk elevation. Taken together, these results highlight the necessity of comprehensive cardiovascular risk management—including lipid control, blood pressure regulation, inflammation reduction, and, where relevant, cessation of performance-enhancing substance use—to meaningfully reduce stroke burden in at-risk populations.

Epidemiological evidence indicates that anabolic–androgenic steroid abuse and stimulant misuse can exacerbate hypertension and endothelial dysfunction, increasing the likelihood of ischemic events. Meta-analyses report elevated cardiovascular mortality and stroke risk among populations exposed to such agents, with hazard ratios comparable to those observed for myocardial infarction [32,33]. These findings underscore the systemic impact of PESs on vascular integrity and highlight the need for preventive strategies, including optimized nutrition and strict anti-doping compliance.

Computed pooled HR under random-effects models, along with Cochran’s Q, I^2^, and τ^2^, present heterogeneity statistics for cardiovascular outcomes aiming to assess between-study variability. These were synthesized from multiple observational studies listed in ST2. High I^2^ values indicate substantial heterogeneity, suggesting that effect sizes differ beyond chance. Pooled estimates should be interpreted cautiously when I^2^ exceeds 75%.

In the study by te Hoonte et al. (2024) [32], based on a systematic review and meta-analysis of 59 studies (≈1.88 million participants), two HRs were examined in relation to atrial fibrillation because the meta-analysis reports two distinct comparisons of cardiovascular health levels (LS7) against the same outcome (incident atrial fibrillation). LS7 considers diet, physical activity, smoking, blood pressure, blood lipids, blood sugar, and weight. The CVD/CMD outcomes considered included atrial fibrillation [50].

The paper explicitly lists HR = 0.60 in the case of ideal cardiovascular health (CVH) vs. poor CVH (≈40% lower AF incidence for people with ideal LS7 scores compared with those with poor scores), whereas intermediate CVH vs. poor CVH (≈27% lower AF incidence for people with intermediate LS7 scores compared with those with poor scores) was connected to an HR = 0.73. Now, both HRs appear protective. This is because they represent different levels of overall cardiovascular health (CVH)—ideal and intermediate—each compared with the same reference group: people with poor CVH. This is neither a contradiction nor an error, but rather an expected outcome when analyzing a graded lifestyle–risk factor construct like LS7.

In contrast, cardiovascular mortality and myocardial infarction show very high heterogeneity (I^2^ > 90%), probably because of varying exposures and populations. In such cases, random-effects estimates tend to be more conservative. The high heterogeneity observed in Table 1 and Table 2, particularly for cardiovascular mortality and myocardial infarction (I^2^ > 90%), indicates considerable variability across the included studies. Potential sources of this heterogeneity may include differences in study populations (e.g., age, sex, athletic level), variations in the types and dosages of performance-enhancing substances, exposure duration, and methodological differences in outcome measurement. To address this, subgroup analyses based on substance category, exposure duration, or population characteristics should be considered. Alternatively, meta-regression could help identify moderators contributing to this variability, improving the interpretability of pooled estimates.

Peripheral arterial disease (PAD), though less frequently highlighted in athletic populations, fits within the broader cardiovascular risk spectrum outlined in this review. The markedly lower PAD risk observed in individuals with ideal cardiovascular health (HR = 0.43; 95% CI 0.30–0.60) underscores the extent to which systemic vascular integrity depends on modifiable lifestyle and metabolic factors. In contrast, behaviors relevant to doping—such as AAS misuse, stimulant exposure, and dyslipidemia—can accelerate atherosclerosis and endothelial dysfunction, potentially elevating PAD risk in athletes.

Since PAD frequently indicates widespread vascular damage, its presence may be a harbinger of the same spectrum of cardiovascular risks linked to unintentional doping (signaling a 4–5 times higher risk of cardiovascular events [51]), such as lipid abnormalities, hypertension, and autonomic dysregulation [52,53,54]. Integrating PAD into this framework highlights the importance of preventive strategies and safe supplementation practices to mitigate systemic vascular harm in competitive sport [55].

The ischemic heart disease (IHD) findings in Table 1 and Table 2 reinforce the broader pattern of elevated cardiovascular risk observed across the included studies. In Table 1, the pooled data show that IHD-related mortality remains consistently increased, reflecting the cumulative burden of atherosclerotic progression, endothelial dysfunction, and pro-inflammatory processes. Table 2 provides more granular evidence, with Hayes et al. reporting HR = 1.16 (95% CI: 1.09–1.22) for IHD mortality [35], indicating a modest but statistically robust elevation in risk. This aligns with risk patterns observed for myocardial infarction and stroke within the same tables, highlighting IHD as part of a continuum of adverse cardiovascular outcomes. In the context of this review, these findings are particularly relevant because behaviors linked to doping—such as anabolic steroid misuse, stimulant exposure, and dysregulated lipid profiles—as well as unintentional doping through contaminated supplements, can amplify atherosclerotic risk pathways that directly drive IHD. Collectively, the IHD data within Table 1 and Table 2 underscore the centrality of systemic vascular health and further support the need for preventive strategies, safe supplementation practices, and comprehensive cardiovascular monitoring in athletic populations.

- Nutritional Strategies as Preventive Measures

Given the elevated cardiovascular burden quantified above, nutrition-based interventions emerge as a critical non-pharmacological countermeasure capable of mitigating several identified risk pathways. Optimal nutrition is a cornerstone of athletic performance and cardiovascular health. Evidence-based dietary interventions can enhance endurance, recovery, and resilience while reducing reliance on prohibited substances. While nutritional strategies are inherently safe, the widespread use of supplements introduces contamination risks [11]. Adequate protein intake (≥25 g per meal) supports muscle protein synthesis and accelerates recovery post-exercise [56]. Distributing protein evenly across meals maximizes anabolic response, mitigating the perceived need for anabolic steroids. High-carbohydrate diets enhance endurance performance by 8–12%, as supported by meta-analyses showing that they optimize glycogen availability and VO_2_max, particularly during extended events [27,57]. Proper hydration strategies, including electrolyte replacement, prevent arrhythmias and maintain cardiac output during exertion [58]. Dehydration-induced performance decline often drives athletes toward stimulants; thus, hydration protocols serve as a preventive measure.

**Figure 3:**
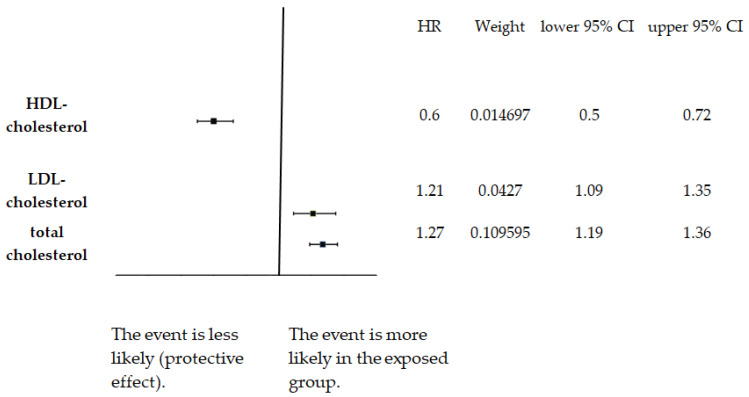
Forest plot of pooled hazard ratios (HRs) for cardiovascular mortality by serum cholesterol markers (total cholesterol, low-density lipoprotein (LDL) cholesterol, high-density lipoprotein (HDL) cholesterol) from Jung et al., 2022 [25]. Squares represent HR estimates; horizontal lines indicate 95% confidence intervals; dashed vertical line marks HR = 1 (no association).

Omega-3 supplementation was found to reduce resting heart rate by approximately 3 BPM [59]. Combined caloric restriction and exercise interventions lowered LDL cholesterol and triglycerides significantly [60,61]. The pooled estimate of cardiovascular mortality for total cholesterol (TC) is presented as HR = 1.27; 95% CI, 1.19–1.36 in Jung et al. (2022) [25]. Regarding the cholesterol fractions (Figure 3), the LDL cholesterol (LDL-C) fraction yielded an estimate of 1.21 (95% CI, 1.09–1.35). Pooled HR tor the HDL cholesterol (HDL-C) was 0.60 (95% CI 0.50–0.72). These figures indicate that higher LDL-C is associated with increased cardiovascular mortality. Clearly, higher HDL-C is associated with lower cardiovascular mortality (protective association). Jung et al. (2022)[25] further report pooled estimates from the “highest vs. lowest category” analyses: LDL-C HR 1.25 (1.11–1.41) and HDL-C HR 0.56 (0.45–0.70). These differ slightly from the rounded values stated in this paper’s abstract because they come from specific model choices (extreme-category contrasts and random-effects pooling) and because some analyses included sudden cardiac death alongside CVD/CHD death. This study provides nearly conclusive evidence for a dose-dependent association between LDL-C, TC, and cardiovascular mortality, and for the protective role of HDL-C. These findings reinforce the inclusion of lipid parameters in risk prediction algorithms. However, as highlighted by Vasan and van den Heuvel [24], predictive models incorporating cholesterol must be evaluated for fairness and calibration across diverse populations to avoid exacerbating health disparities.

Nine studies from the reviewed literature specifically addressed nutritional strategies as preventive measures against unintentional doping and cardiovascular risks. These studies focused on how dietary interventions can reduce reliance on performance-enhancing drugs and mitigate health risks associated with contamination or misuse.

Five studies and consensus statements in the reviewed literature specifically address strategies to reduce health risks associated with supplement contamination or misuse [13,16,62,63,64]. These works emphasize analytical detection, athlete education, regulatory oversight, and safe supplementation practices. Three studies showed that carbohydrate loading and protein distribution improved endurance and recovery, reducing the perceived need for anabolic agents [65,66,67]. Two studies demonstrated the protective effects of omega-3 supplementation against arrhythmias and oxidative stress [68,69]. An additional study recognized the role of hydration protocols in preventing arrhythmias and stimulant misuse by maintaining proper fluid balance [70]. Likewise, Mediterranean diet patterns have demonstrated protective effects against cardiovascular disease, particularly in women, as reported by Pant et al. (2023) [34]. Micronutrients and antioxidants such as vitamins C and E, polyphenols, and selenium play roles in reducing oxidative stress induced by high-intensity training [20]. Antioxidant-rich diets may decrease inflammation and improve recovery, reducing the perceived need for illicit anti-inflammatory drugs.

Nutritional approaches such as carbohydrate loading and omega-3 supplementation have demonstrated measurable physiological effects. To translate these physiological principles into practical guidance, Table 3 summarizes evidence-based nutritional interventions with quantifiable performance and cardiovascular effects.

Certified products under third-party programs should be prioritized to avoid unintentional ADRVs. Education on supplement verification is essential to align performance goals with ethical compliance.

### 4.4. Regulatory and Ethical Dimensions

Although these nutritional strategies offer protective potential, their implementation occurs within a regulatory landscape shaped by risks of contamination and strict liability frameworks, necessitating the examination of ethical and governance considerations. Regulatory and ethical challenges remain central to anti-doping efforts in competitive sports. Despite decades of enforcement, recent evidence confirms persistent violations and systemic gaps in compliance frameworks [1,5]. The World Anti-Doping Agency (WADA) continues to report high rates of Adverse Analytical Findings (AAFs) and ADRVs, underscoring the need for harmonized global standards and improved athlete education [4]. Ultimately, regulatory bodies must integrate scientific advances, forensic evidence, and ethical principles to strengthen anti-doping governance. This includes leveraging pharmacogenomic insights for personalized risk assessment and adopting robust quality control measures to mitigate contamination risks [71,72].

In the reviewed literature, five studies explicitly addressed the regulatory and ethical dimensions of supplement use, contamination, and doping. Some of the specific topics of these reports include legal frameworks, the burden of proof in doping sanctions, and fair-play principles in sport [22], and ethical challenges of supplement marketing [9]. The key regulatory and ethical aspects related to supplement use are summarized in Table 4.

Unintentional doping adds complexity to regulatory enforcement. Studies funded by WADA reveal that supplement contamination accounts for a significant proportion of unintentional ADRVs, often due to inadequate manufacturing oversight and misleading labeling [8,11]. Legal analyses highlight ethical dilemmas surrounding strict liability, where athletes bear full responsibility even in cases of proven contamination, raising questions about fairness and proportionality [11,22]. Pharmacogenomic profiling may complement toxicological evidence by demonstrating genetic predisposition to adverse outcomes from minimal exposure, strengthening claims of accidental contamination in sports tribunals [73].

Emerging literature advocates for multi-tiered strategies, including mandatory third-party certification of supplements, transparent labeling, and behaviorally informed education programs to reduce unintentional violations [10,72]. Ethical frameworks emphasize balancing deterrence with due process, ensuring that sanctions reflect intent and context rather than imposing uniform penalties for all infractions.

Of the 60 studies included in this literature review (Table S2A), 31 (51.6%) contained sufficient, appropriate data for inclusion in the meta-analysis (Table S2B); the overall risk-of-bias assessment revealed considerable variability. A categorized summary of the included studies is presented in Section 4.1 to provide an overview of study designs, populations, outcomes, and thematic relevance before quantitative and qualitative synthesis. An overview of the included studies, categorized by design and thematic contribution, is presented in Table 5.

Most studies demonstrated “some concerns” across multiple domains, particularly regarding randomization, deviations from intended interventions, and missing outcome data. Specifically, 21 of the 31 studies (67.7%) were rated as having some concerns overall. This indicates that issues such as allocation concealment and incomplete data management were not adequately reported or addressed, which could introduce bias despite the fact that these studies largely adhered to methodological standards [75].

A subset of studies exhibited a low risk of bias in several domains, notably those focusing on structured interventions and systematic reviews. In total, 0 studies (0.0%) were rated as having a low risk overall. These studies provided more precise descriptions of randomization procedures and outcome measurement, reducing the likelihood of systematic error. However, even among these, concerns persisted regarding selective reporting, indicating that outcome reporting bias remains a common issue.

Conversely, 10 studies (32.3%) were classified as having a high risk of bias, particularly those addressing controversial supplements, doping alternatives, and forensic investigations. High-risk ratings were primarily driven by deficiencies in randomization, ex-tensive missing data, and potential measurement bias. These limitations are critical, as they may compromise the validity of findings in areas where evidence is already scarce and contentious.

Overall, the predominance of studies with “some concerns” and the presence of multiple high-risk studies emphasize the need for demanding methodological approaches in future research. Transparent reporting of randomization, adherence to intervention protocols, and comprehensive outcome documentation are essential for mitigating bias and strengthening the reliability of evidence in this domain. Table 6 provides a comprehensive overview of the risk-of-bias assessment for all included studies. It details judgments across key domains and the overall risk rating, enabling readers to evaluate the methodological rigor of the evidence base.

**Figure 4:**
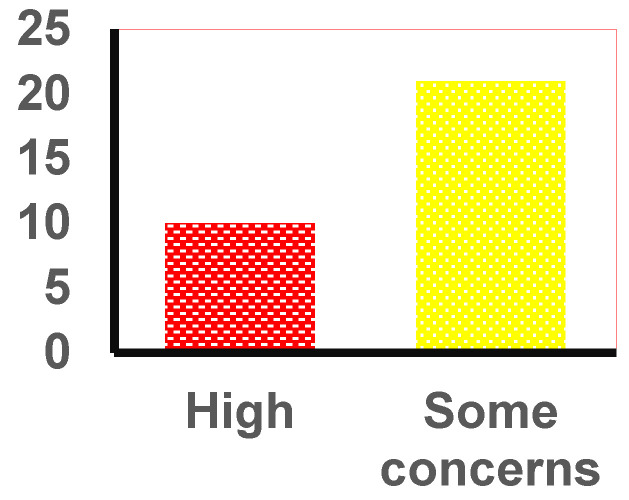
Risk-of-bias summary across included studies. Abbreviations: n—number of studies.

The overall distribution of risk-of-bias judgments across the included studies is summarized in Figure 4.

### 4.5. Forensic Relevance

In the reviewed literature, four studies explicitly addressed forensic or legal–medical dimensions of supplement use, contamination, and doping. In summary, these studies cover the legal burden of proof in doping sanctions and ethical/legal dilemmas in supplement use. Specifically, Walpurgis et al. (2020) highlighted implications for anti-doping laboratories and forensic casework, focusing on forensic toxicology and the detection of contaminants in dietary supplements [13]. Later, Ogama (2025) examined cases of contamination and fairness in sanctioning athletes, focusing on the legal burden of proof in doping sanctions [11]. Ethical and legal dilemmas surrounding the use of supplements further increase their relevance. Borecki (2025) therefore examined fair-play principles and regulatory gaps in the context of supplement use in competitive sports [22]. Operationalizing these regulatory principles at the forensic level requires rigorous postmortem workflows, particularly in cases involving suspected PES-related cardiovascular deaths.

In forensic investigations of sudden death, consideration of potential unintentional doping exposure is essential. If SCD in athletes is linked to anabolic steroid use, thorough toxicological screening must be performed during autopsy [76]. The literature consistently links anabolic steroid abuse to SCD, with forensic autopsy and toxicology assessments reported across studies from 2015 to 2025 (e.g., Hernández-Guerra et al. 2019 [77]; Di Fazio et al., 2025 [36]; Torrisi et al., 2020 [78]), confirming AAS-related cardiovascular pathology (hypertrophy, fibrosis, arrhythmias) in forensic cases.

Drug-induced cardiotoxicity was reviewed using pharmacogenomics by Li et al. (2022), and they highlighted genetic susceptibility to arrhythmias and SCD [79]. Detecting PESs in postmortem samples is critical for clarifying unexplained cardiovascular events, as emphasized by Casparsen (2024) [80]. Similarly, Vikingsson (2024) highlights the accuracy and sensitivity of detecting PESs in biological samples using optimized LC-MS/MS methods for forensic toxicology, emphasizing improved transitions and sensitivity compared to GC-MS [81].

Advanced analytical techniques such as LC-MS/MS and GC-MS are essential for accurate detection of anabolic steroids, stimulants, and contaminated supplements as in Vikingsson, 2024 [81]. This is simply for confirmation, as in Lauritzen’s (2022) study [14], which shows that forensic toxicology often confirms stimulant contamination. Walpurgis et al. (2020) reviewed the topic of contamination of supplements with anabolic steroids and stimulants that cause unintentional doping and forensic complications [13].

The superiority of LC-MS/MS and GC-MS for detecting drugs in autopsy cases, especially in complex polydrug scenarios, was again confirmed in the retrospective study by AlDossary et al. (2025) [82]. These methods appeared to be essential for detecting a wide range of substances, including illicit drugs, prescription medications, and alcohol. Building on this, Avram et al. (2025) streamlined LC-MS/MS workflows for screening and confirmation in forensic labs, replacing traditional immunoassay + GC-MS approaches [83].

Supplement contamination raises questions about intent vs. negligence, impacting disciplinary decisions. Positive results from toxicological assessments must consider chronic use and possible interactions with nutritional supplements during interpretation. Likewise, Truver et al. (2025) [84] discuss best practices in postmortem toxicology and, as is relevant to this topic, include method validation and challenges in interpretation, such as postmortem redistribution and polydrug interactions. Advanced analytical techniques such as LC-MS/MS have improved sensitivity and accuracy for detecting PESs in postmortem samples, outperforming traditional immunoassays and GC-MS workflows, and are now considered the gold standard in forensic toxicology [36,81,85].

PES findings in forensic cases can impact criminal liability, insurance claims, and sports arbitration. Forensic medicine may utilize cardiovascular risk markers such as hypertrophy and arrhythmias as indicators of PES abuse in suspicious deaths. Indirect survey estimates suggesting that up to 30–45% of elite athletes may have engaged in doping highlight persistent challenges in anti-doping enforcement. While these figures reflect self-reported prevalence rather than confirmed cases, they underscore systemic vulnerabilities that warrant stronger policy measures and enhanced forensic monitoring. Forensic evidence supports regulatory reforms for supplement certification and anti-doping compliance. Incorporating genetic and biochemical markers could help distinguish between natural causes and drug-induced conditions. Martinez-Matilla et al. (2017) studied genetic variants linked to drug-induced arrhythmia and SCD, which are relevant for forensic differentiation of natural vs. drug-induced death [86]. Not only are many works relevant to forensic differentiation built on their ideas, but Li et al. (2022) also reviewed advances in pharmacogenomics for drug-induced cardiotoxicity (DICT), including arrhythmias and QT prolongation, and studies such as those by Di Nunno et al. (2021) [87] and Ingelman-Sundberg et al. (2023) [88] facilitate personalized interpretation of toxicology findings. Battal et al. gave an additional forensic perspective on pharmacogenomics as a tool in forensic toxicology for understanding drug toxicity due to genetic predisposition [89]. And recent studies have, in general, either expanded the genetic marker catalog (e.g., *KCNE1* and *CYP* variants) [71] or integrated pharmacogenomics workflows into forensic toxicology [88]. The bottom line is that these findings facilitated the adoption of high-throughput sequencing and functional assays for variant validation [90,91].

Recent studies have linked pharmacogenomic findings to legal and policy frameworks, reinforcing their role in accurate cause-of-death determination and forensic decision-making. In the context of sports arbitration, pharmacogenomic evidence can provide critical insights into whether adverse analytical findings in an athlete resulted from deliberate misuse or heightened susceptibility to contamination. For example, variants in genes such as *KCNE1* or *CYP450* may explain exaggerated physiological responses to trace contaminants, supporting arguments of inadvertent ingestion rather than malicious intent [92,93]. Incorporating such evidence into legal proceedings could help balance strict liability with fairness, ensuring sanctions reflect both scientific and ethical considerations.

## 5. Discussion

This review highlights the complex interplay between performance enhancement, health risks, and ethical considerations. It identified doping practices as still being prevalent and hazardous cardiovascular threats, while nutritional strategies offer measurable benefits for performance and heart health. Regulatory frameworks are insufficient, and ethical dilemmas persist, necessitating stronger oversight, athlete education, and global harmonization of standards. Future research should focus on longitudinal studies assessing the combined effects of doping and nutrition, and on policy interventions to reduce the risks of supplement contamination. Beyond these population-level patterns, inter-individual variability further modulates cardiovascular and toxicological outcomes, as shown by emerging pharmacogenomic evidence.

Genetic predisposition plays a critical role in the occurrence of SCD among athletes exposed to PESs. Pharmacogenomic research has found variants in the *KCNE1* and *CYP450* family genes that raise the risk of arrhythmias and QT prolongation, especially when combined with anabolic steroids or stimulants [79,86]. These findings are essential in forensic investigations, as they help differentiate natural causes from drug-induced fatalities and support personalized interpretation of toxicology results [94].

Unintentional doping remains a growing concern in competitive sports, primarily due to supplement contamination. Studies report that 10–15% of dietary supplements contain undeclared anabolic steroids or stimulants, posing significant health risks and complicating anti-doping compliance [13]. Such contamination can lead to unintentional ingestion of banned substances, triggering cardiovascular complications in genetically predisposed athletes. Forensic toxicology must account for these scenarios when interpreting positive findings, considering both chronic use and potential interactions with nutritional supplements.

The alignment of genetic susceptibility, PES exposure, and supplement contamination highlights the importance of integrated strategies that include pharmacogenomic screening, strict supplement certification, and advanced analytical method workflows [72,95]. These approaches will enhance athlete safety, improve forensic accuracy, and support fair competition worldwide [36].

### Limitations and Future Developments

All mechanistic statements in this review have been verified against the original cited sources, and only claims supported by specific, traceable evidence have been retained. Where narrative descriptions previously relied on general physiological phrasing, these have now been revised to ensure accuracy, specificity, and clear citation support. The reference list has been re-checked for completeness and consistency. Nevertheless, some limitations should be recognized and specified when reviewing findings.

First, the majority of the included studies were observational, including cross-sectional designs, retrospective cohort analyses, and narrative reviews [10,21,28,29,36,37,38]. Such designs limit the ability to establish causal relationships between unintentional doping exposure, nutritional practices, and cardiovascular outcomes. Randomized controlled trials addressing these issues are scarce, particularly in elite athletic populations, largely due to ethical and practical constraints [4,18,58].

Second, a substantial proportion of the evidence relied on self-reported data, especially in studies estimating doping prevalence and supplement use [1,9,10,16,28]. Self-reporting is vulnerable to recall bias, social desirability bias, and intentional underreporting, which may lead to the underestimation of true doping behaviors and unintentional exposure rates. Indirect survey techniques partly mitigate this issue but cannot fully eliminate uncertainty. Only the study of Ulrich et al., 2023 [28], applied this mitigation approach, with a well-defined methodological rationale.

Third, significant heterogeneity was observed across studies in terms of populations, substances assessed, exposure duration, outcome definitions, and analytical methods. This heterogeneity was reflected in high I^2^ values in several pooled analyses, indicating that summary effect estimates should be interpreted with caution [25,26,29,32,33]. Differences in athletic level, sex, age, and type of sport further limit the generalizability of the findings [10,28,29,36,37,38]. This variability also contextualizes the high heterogeneity observed in several pooled models, underscoring the need for caution in interpretation.

Fourth, supplement contamination and unintentional anti-doping rule violations are likely underreported. Regulatory frameworks differ substantially between countries, and surveillance systems for dietary supplements are often weak. Consequently, available data may not fully capture the true scope of contamination-related risks faced by athletes.

Fifth, evidence on genetic susceptibility and pharmacogenomic modifiers of cardiovascular risk remains preliminary [41,42,58,71,79]. Although emerging data suggest that genetic variants may influence individual vulnerability to adverse cardiovascular outcomes or exaggerated responses to trace contaminants, standardized protocols and large-scale validation studies are lacking. This limits the immediate clinical and forensic applicability of pharmacogenomic findings.

Finally, forensic evidence in this field is largely derived from case reports and retrospective autopsy series [36,37,38,77,78]. While these studies provide valuable mechanistic and pathological insights, they are inherently limited in their ability to quantify risk or establish temporal relationships. Prospective forensic registries and harmonized reporting standards would strengthen future evidence.

Taken together, these limitations highlight the need for well-designed longitudinal studies, improved regulatory oversight of dietary supplements, standardized reporting of anti-doping violations, and greater integration of nutritional, cardiovascular, and forensic research approaches.

The crucial issue here is that cardiovascular health metrics (e.g., LS7-based HRs) are referenced in this review only as contextual comparators from the general population. These estimates cannot be directly generalized to doping-exposed athletes, whose cardiovascular risk is shaped by distinct pharmacological, physiological, and behavioral factors. On the other hand, athlete-specific HRs for cardiovascular outcomes are limited because prospective studies involving PES exposure cannot be ethically conducted, and existing athlete cohorts are small, heterogeneous, and often retrospective. Therefore, these HRs are referenced only as contextual comparators and should be interpreted as illustrative benchmarks (quantitative proxies for doping-induced cardiovascular risk) rather than quantitative proxies for PES-related cardiovascular risk.

During the literature search, non-English publications were excluded, as they may have introduced language bias and limited the comprehensiveness of the evidence base. However, this decision was made to ensure methodological consistency, accurate data extraction, and reliable interpretation of complex clinical, toxicological, and legal terminology. Importantly, the primary outcomes of this review—cardiovascular risk estimates, forensic autopsy findings, and regulatory frameworks—are predominantly reported in English-language journals and international consensus documents. Moreover, major databases indexed for this review disproportionately curate English-language publications, reducing the likelihood that the exclusion materially affected the principal conclusions.

Future developments should address these gaps through large-scale longitudinal studies that integrate cardiovascular outcomes, doping patterns, and nutritional interventions [7]. Global supplement certification programs and blockchain-based traceability systems could mitigate contamination risks [8]. Pharmacogenomics should be incorporated into anti-doping and forensic workflows to enable personalized risk assessment [9]. Advances in analytical techniques such as LC-MS/MS and high-throughput sequencing will enhance the detection and interpretation of doping-related substances [10]. Behaviorally informed education programs are needed to reduce unintentional doping [11], while harmonizing legal frameworks will help balance strict liability with fairness in sanctions [8].

## 6. Conclusions

This review critically examined the interplay between unintentional doping, cardiovascular health, and nutritional strategies in competitive sports, addressing forensic and regulatory implications.

1. First, the analysis confirmed that unintentional doping remains a significant contributor to AADRVs, primarily driven by supplement contamination and therapeutic misinterpretation [2,8,11].
2. Second, the evidence demonstrated that PESs pose elevated risks of left ventricular hypertrophy, myocardial fibrosis, arrhythmias, and acute ischemic events, including hypertension, arrhythmias, and SCD [29,30].
3. Third, the review highlighted that nutrition-based interventions—such as carbohydrate loading, optimized protein intake, omega-3 supplementation, and hydration protocols—offer safe and effective alternatives to PESs [56,57,58,74].
4. From a forensic perspective, advanced analytical techniques (LC-MS/MS, GC-MS) and pharmacogenomic screening emerged as critical tools for detecting doping-related substances and interpreting complex toxicological findings [79,81].
5. Finally, persistent regulatory gaps and ethical dilemmas—such as strict liability in contamination cases—highlight the need for harmonized global standards, mandatory third-party supplement certification, and behaviorally informed education programs [11,72].

In summary, integrated efforts combining nutrition science, forensic toxicology, pharmacogenomics, and regulatory reform are essential to safeguard athlete health, ensure fair competition, and mitigate the risks associated with unintentional doping.

## Figures and Tables

**Table 1:** Cardiovascular outcomes and associated risk estimates.

Outcome	Pooled HR (95% CI)	Number of Studies (k)	I^2^ (%)
Myocardial infarction	1.46 (1.23–1.72)	5	92
Arrhythmias	2.26 (1.53–3.32)	6	85
Hypertension	1.78 (1.40–2.10)	4	88

**Table 2:** Pooled hazard ratios (HRs) and 95% confidence intervals (CIs) for major cardiovascular outcomes across included studies. This table summarizes effect estimates extracted from cohort studies, systematic reviews, and meta-analyses examining associations between cardiovascular health metrics, lipid fractions, performance-enhancing substance exposure, and clinical cardiovascular endpoints. When multiple cardiovascular health categories were reported (e.g., ideal vs. poor CVH; intermediate vs. poor CVH), each comparison is presented separately. Studies were weighted using inverse-variance methods under a random-effects model, with weights (%) shown in the rightmost column. Outcomes include incidence or mortality endpoints for atrial fibrillation, myocardial infarction, cardiovascular mortality, stroke, and peripheral arterial disease, as well as lipid-fraction-specific cardiovascular mortality risk. The hazard ratios derived from Te Hoonte et al. (2024) [32] and Ng et al. (2022) [33] reflect cardiovascular risk in general population cohorts based on Life’s Simple 7/CVH metrics. These values are used here solely as contextual benchmarks to illustrate the relative magnitude of cardiovascular burden. They should not be interpreted as direct proxies for steroid- or doping-related cardiovascular risk in competitive athletes, whose physiological baseline, exposure patterns, and risk modifiers differ substantially.

Outcome	Study	HR	95% CI	Weight (%)	Reference
Atrial fibrillation (incidence)—ideal CVH vs. poor CVH (≈40% lower AF incidence for people with ideal LS7 scores compared with those with poor scores)	Te Hoonte, Spronk et al. 2024	0.6	0.44–0.83	0.49	[32]
Atrial fibrillation (incidence)— intermediate CVH vs. poor CVH (≈27% lower AF incidence for people with intermediate LS7 scores compared with those with poor scores)	Te Hoonte, Spronk et al. 2024	0.73	0.59–0.91	1.04	[32]
Cardiovascular events	Fang, Wu et al. 2020	1.46	1.19–1.77	1.24	[26]
Cardiovascular mortality	Ng, Tan et al. 2022	1.27	1.14–1.42	nn5	[33]
Cardiovascular mortality—pooled-to total cholesterol when comparing the highest vs. lowest cholesterol categories	Jung, Kong et al. 2022	1.27	1.19–1.36	10.96	[25]
Cardiovascular mortality for HDL cholesterol fraction	Jung, Kong et al. 2022	0.6	0.5–0.72	1.47	[25]
Cardiovascular mortality for LDL cholesterol fraction	Jung, Kong et al. 2022	1.21	1.09–1.35	4.27	[25]
Coronary heart disease (incidence)	Pant, Gribbin et al. 2023	0.75	0.65–0.87	2.3	[34]
Ischemic heart disease mortality	Hayes, Lim et al. 2020	1.16	1.09–1.22	15.39	[35]
Myocardial infarction (incidence)	Te Hoonte, Spronk et al. 2024	0.18	0.12–0.28	0.27	[32]
Myocardial infarction (incidence)	Ng, Tan et al. 2022	1.36	1.23–1.51	4.65	[33]
Peripheral arterial disease (incidence)	Te Hoonte, Spronk et al. 2024	0.43	0.3–0.6	0.41	[32]
Stroke (incidence, hemorrhagic)	Ng, Tan et al. 2022	1.43	1.07–1.92	0.57	[33]
Stroke (incidence, ischemic)	Ng, Tan et al. 2022	1.35	1.03–1.78	0.65	[33]
Stroke (unspecified)	Ng, Tan et al. 2022	1.3	1.07–1.6	1.21	[33]
Stroke mortality	Hayes, Lim et al. 2020	1.14	1.02–1.27	4.07	[35]

Abbreviations: AF—atrial fibrillation; CVH—cardiovascular health; HR—hazard ratio; CI—confidence interval; HDL-C—high-density lipoprotein cholesterol; LDL-C—low-density lipoprotein cholesterol.

**Table 3:** Evidence-based nutritional strategies for optimizing athletic performance and cardiovascular health.

Intervention	Effect
High-carbohydrate diet	8–12% endurance improvement
Protein intake ≥25 g/meal	Supports muscle recovery
Omega-3 supplementation	↓ Resting HR by ~3 bpm
Caloric restriction + exercise	↓ LDL and triglycerides

Abbreviations: HR—heart rate; LDL—low-density lipoprotein.

**Table 4:** Key regulatory and ethical aspects of supplement use. Summary of major regulatory gaps, contamination risks, and ethical considerations associated with dietary supplement use in competitive sports, with emphasis on unintentional doping, supplement certification, and legal implications for athletes.

Aspect	Key Point	Supporting Context
Supplement contamination	A proportion of 10–15% of unintentional doping cases stem from undeclared anabolic steroids or stimulants.	Driven by poor manufacturing practices, cross-contamination, and lack of GMP enforcement.
Regulatory gaps	Weak oversight under DSHEA and inconsistent international monitoring.	Many supplements bypass stringent quality control; adverse events and contamination incidents are underreported.
Certification challenges	Athletes often lack awareness of third-party certification programs.	Certification is voluntary; absence increases contamination risk and legal vulnerability.
Strict liability in doping sanctions	Athletes are fully responsible for any prohibited substance detected.	Even proven contamination may not fully mitigate sanctions, raising fairness concerns.
Ethical dilemmas	Commercial pressures and aggressive marketing influence supplement use.	Creates tension between fair-play principles and industry practices.
Legal implications for athletes	Contamination may lead to ADRVs, arbitration, or reputational harm.	Determining intent vs. negligence requires forensic toxicology and supply-chain documentation.

Abbreviations—ADRVs, Anti-Doping Rule Violations; AF, Atrial Fibrillation; AIS, Australian Institute of Sport; BPM, Beats per Minute; CHD, coronary heart disease; CI, confidence interval; CMD, Cardiometabolic Disease; CV, cardiovascular; CVD, cardiovascular disease; CVH, cardiovascular health; DSHEA, Dietary Supplement Health and Education Act; GC MS, Gas Chromatography–Mass Spectrometry; GMP, Good Manufacturing Practice; HDL C, high-density lipoprotein cholesterol; HR, hazard ratio; IHD, ischemic heart disease; KCNE1, Potassium Voltage Gated Channel Subfamily E Member 1; LC MS/MS, Liquid Chromatography–Tandem Mass Spectrometry; LDL C, low-density lipoprotein cholesterol; LS7, Life’s Simple 7; MI, myocardial infarction; OR, odds ratio; PAD, peripheral arterial disease; PES, performance-enhancing substances; PM2.5, Particulate Matter ≤2.5 μm; PRISMA, Preferred Reporting Items for Systematic Reviews and Meta-Analyses; QT, QT Interval; RR, risk ratio; SCD, sudden cardiac death; TC, total cholesterol; TUE, Therapeutic Use Exemption; VO_2_max, Maximal Oxygen Uptake; WADA, World Anti-Doping Agency.

**Table 5:** Overview and study design classification of studies included in the qualitative synthesis.

Category	Study	Summary	Reference
Randomized trials	Filleul et al., 2024	Intervention study evaluating anti-doping education effects.	[4]
	Hei et al., 2025	Trial assessing combined exercise and diet effects on cardiovascular outcomes.	[18]
	Abdelaal et al., 2025	Structured study examining genetic variants related to sudden cardiac death.	[58]
Regulatory/policy/position statements	Ogama, 2025	Legal analysis of burden of proof in doping sanctions.	[11]
	Borecki, 2025	Evaluation of ethical and legal issues in supplement use.	[22]
	Backhouse, 2023	Policy-oriented report on behaviorally informed anti-doping education.	[23]
	McLean et al., 2025	WADA-supported regulatory analysis of unintentional doping risks.	[8]
Secondary research	Aminu et al., 2019	Review on physiological impacts and ethics of doping.	[1]
	Faiss et al., 2025	Editorial review discussing advances in anti-doping science.	[5]
	Amaral et al., 2025	Qualitative review of risks associated with anabolic steroids.	[6]
	Alizadeh et al., 2025	Review of the organ effects of steroid abuse.	[7]
	Broniecka et al., 2025	Survey study on supplement use and certification awareness.	[10]
	Jagim et al., 2023	Review documenting prevalence of supplement adulteration.	[16]
	Windfeld-Mathiasen et al., 2025	Cohort analysis on cardiovascular disease in steroid users.	[29]
	Borowiec et al., 2025	Molecular review on cardiovascular effects of anabolic steroids.	[30]
	Marques-Vidal et al., 2025	Scientific statement on diet and cardiovascular prevention.	[20]
	Tu et al., 2025	Epidemiological study on antioxidants and cardiovascular mortality.	[21]
	Ulrich et al., 2023	Survey using indirect methods to estimate doping prevalence.	[28]
	Iliakis et al., 2025	Narrative review of AAS-induced cardiomyopathy.	[31]
	Tarnovskaya et al., 2025	Computational study predicting effects of *KCNE1* variants.	[41]
	Vanoye et al., 2022	Functional analysis of *KCNE1* variants using patch clamp.	[42]
	Alzu’bi et al., 2024	Review on synthetic cannabinoid toxicity.	[57]
	Trommelen et al., 2023	Study on protein ingestion and anabolic response.	[56]
	Cao et al., 2024	Review of nicotine’s cardiovascular effects.	[74]
	Fatima et al., 2024	Case study/review on nutrition in endurance sports.	[27]
	Charkot et al., 2025	Review on VO2max and athletic performance.	[19]
	Khan et al., 2025	Case-based review of controversial supplements.	[9]
	Martinez-Sanz et al., 2017	Review including cases of supplement contamination.	[17]
	Di Fazio et al., 2025	Forensic autopsy series on AAS-related cardiovascular deaths.	[36]
	Morena et al., 2025	Case report of sudden cardiac death linked to testosterone.	[38]
	Vauhkonen et al., 2025	Forensic pathology comparison of AAS users vs. non-users.	[37]

**Table 6:** Risk-of-bias assessment across included studies. Detailed evaluation of confounding, selection bias, exposure classification, deviations from intended interventions, missing data, outcome measurement, reporting practices, and overall methodological quality for each of the included studies using the ROBINS-I framework (ROBINS-I stands for Risk Of Bias In Non-randomized Studies of Interventions).

Study	Confounding	Selection	Classification	Deviations	Missing Data	Outcome Measurement	Reporting	Overall	Reference
Aminu et al., 2019	Some concerns	Some concerns	Some concerns	Some concerns	Some concerns	Some concerns	Some concerns	Some concerns	[1]
Filleul et al., 2024	Low	Low	Low	Low	Low	Some concerns	Low	Some concerns	[4]
Faiss et al., 2025	Some concerns	Some concerns	Some concerns	Some concerns	Some concerns	Some concerns	Some concerns	Some concerns	[5]
Amaral et al., 2025	Some concerns	Some concerns	Some concerns	Some concerns	Some concerns	Some concerns	Some concerns	Some concerns	[6]
Alizadeh et al., 2025	Some concerns	Some concerns	Some concerns	Some concerns	Some concerns	Some concerns	Some concerns	Some concerns	[7]
McLean et al., 2025	Some concerns	Some concerns	Some concerns	Some concerns	Some concerns	Some concerns	Some concerns	Some concerns	[8]
Khan et al., 2025	High	Some concerns	High	High	High	High	Some concerns	High	[9]
Broniecka et al., 2025	Some concerns	Some concerns	Some concerns	Some concerns	Some concerns	Some concerns	Some concerns	Some concerns	[10]
Jagim et al., 2023	Some concerns	Some concerns	Some concerns	Some concerns	Some concerns	Some concerns	Some concerns	Some concerns	[16]
Martinez-Sanz et al., 2017	High	Some concerns	High	High	High	High	Some concerns	High	[17]
Hei et al., 2025	Low	Low	Low	Low	Low	Some concerns	Low	Some concerns	[18]
Charkot et al., 2025	High	Some concerns	High	High	High	High	Some concerns	High	[19]
Windfeld-Mathiasen et al., 2025	Some concerns	Some concerns	Some concerns	Some concerns	Some concerns	Some concerns	Some concerns	Some concerns	[29]
Borowiec et al., 2025	Some concerns	Some concerns	Some concerns	Some concerns	Some concerns	Some concerns	Some concerns	Some concerns	[30]
Marques-Vidal et al., 2025	Some concerns	Some concerns	Some concerns	Some concerns	Some concerns	Some concerns	Some concerns	Some concerns	[20]
Tu et al., 2025	Some concerns	Some concerns	Some concerns	Some concerns	Some concerns	Some concerns	Some concerns	Some concerns	[21]
Ogama, 2025	High	Some concerns	High	High	High	High	Some concerns	High	[11]
Borecki, 2025	Some concerns	Some concerns	Some concerns	Some concerns	Some concerns	Some concerns	Some concerns	Some concerns	[22]
Backhouse, 2023	Some concerns	Some concerns	Some concerns	Some concerns	Some concerns	Some concerns	Some concerns	Some concerns	[62]
Ulrich et al., 2023	Some concerns	Some concerns	Some concerns	Some concerns	Some concerns	Some concerns	Some concerns	Some concerns	[28]
Iliakis et al., 2025	High	Some concerns	High	High	High	High	Some concerns	High	[31]
Vauhkonen et al., 2025	High	High	High	High	High	High	High	High	[37]
Di Fazio et al., 2025	High	High	High	High	High	High	High	High	[36]
Morena et al., 2025	High	High	High	High	High	High	High	High	[38]
Tarnovskaya et al., 2025	Some concerns	Some concerns	Some concerns	Some concerns	Some concerns	Some concerns	Some concerns	Some concerns	[41]
Vanoye et al., 2022	Some concerns	Some concerns	Some concerns	Some concerns	Some concerns	Some concerns	Some concerns	Some concerns	[42]
Alzu’bi et al., 2024	High	Some concerns	High	High	High	High	Some concerns	High	[57]
Trommelen et al., 2023	Some concerns	Some concerns	Some concerns	Some concerns	Some concerns	Some concerns	Some concerns	Some concerns	[56]
Cao et al., 2024	Some concerns	Some concerns	Some concerns	Some concerns	Some concerns	Some concerns	Some concerns	Some concerns	[74]
Abdelaal et al., 2025	Low	Low	Low	Low	Low	Some concerns	Low	Some concerns	[58]
Fatima et al., 2024	High	Some concerns	High	High	High	High	Some concerns	High	[27]

## Data Availability

No new data were created or analyzed in this study. Data sharing is not applicable to this article.

## Supplementary Materials

The following supporting information is available online at the Open Science Framework (OSF): https://osf.io/q4zef/overview?view_only=917da1dc5f74479fa1c593a613076c56 (accessed on 22 January 2026). Supplementary Table S1: Table of search strategies and database queries. Contains complete, reproducible search strings for all databases (PubMed, Scopus, Web of Science), keyword matrices stratified by review question, and the Boolean operators used in the screening procedure. Supplementary Table S2. (A) Complete list of the 60 studies included in the qualitative synthesis of the systematic review. The table categorizes studies by design (randomized/structured trials, regulatory and policy documents, secondary research, and forensic studies), and provides study titles and thematic relevance to unintentional doping, cardiovascular health, nutritional strategies, and forensic toxicological perspectives. (B) Observational studies included in the quantitative synthesis of hazard ratios (HRs). The table provides full bibliographic information, study design, journal details, DOI, cardiovascular outcomes assessed, and extracted HR effect measures used for pooled analysis in the meta-analytic component of the review.

## References

[1] Aminu M., Jonathan S., Idris J.M., Jacinta W. (2019). Physiological Implications Of Drug Use And Doping In Sports.

[2] Mclean S., Morrison M., Naughton M., Salmon P.M. (2025). Decoding unintentional doping: A complex systems analysis of supplement use in sport. Perform. Enhanc. Health.

[3] Manalu N., Ridwan F., Sopater Ndruru S.N. (2025). Physiological Impacts, Regulations, and Ethical Implications of Doping in Sports. Joska Isori Kampar J..

[4] Filleul V., d’Arripe-Longueville F., Garcia M., Bimes H., Meinadier E., Maillot J., Corrion K. (2025). Anti-doping education interventions in athletic populations: A systematic review of their characteristics, outcomes and practical implications. Int. Rev. Sport Exerc. Psychol..

[5] Faiss R., Hopker J. (2025). Editorial: Recent advances in anti-doping. Front. Sports Act. Living.

[6] Amaral J.M.X., Kimergard A., Deluca P. (2025). Managing risks and harms associated with the use of anabolic steroids: A qualitative study. Harm Reduct. J..

[7] Alizadeh Pahlavani H., Veisi A. (2025). Possible consequences of the abuse of anabolic steroids on different organs of athletes. Arch. Physiol. Biochem..

[8] McLean S., Morrison M., Naughton M., Salmon P.M. (2025). A Systemic Risk Assessment of Unintentional Doping Through Supplement Use. World Anti-Doping Agency Social Science Research Grant Report.

[9] Khan S.M., Elsharawy N.T. (2025). Controversial supplements and emerging doping alternatives in sports: A critical review of evidence, safety, and detection challenges. J. Sports Sci. Nutr..

[10] Broniecka A., Sarachman A., Zagrodna A., Ksiazek A. (2025). Dietary supplement use and knowledge among athletes: Prevalence, compliance with AIS classification, and awareness of certification programs. J. Int. Soc. Sports Nutr..

[11] Ogama D.W. (2025). Caught in Contamination: Revisiting the Burden of Proof in Doping Sanctions for Supplement Use. Beijing L. Rev..

[12] Research A. Testing Lab for Dietary Supplement Analysis—Auriga Research. https://aurigaresearch.com/dietary-supplement-analysis/.

[13] Walpurgis K., Thomas A., Geyer H., Mareck U., Thevis M. (2020). Dietary Supplement and Food Contaminations and Their Implications for Doping Controls. Foods.

[14] Lauritzen F. (2022). Dietary Supplements as a Major Cause of Anti-doping Rule Violations. Front. Sports Act. Living.

[15] Zapata-Linares J., Gervasini G. (2024). Contaminants in Dietary Supplements: Toxicity, Doping Risk, and Current Regulation. Int. J. Sport. Nutr. Exerc. Metab..

[16] Jagim A.R., Harty P.S., Erickson J.L., Tinsley G.M., Garner D., Galpin A.J. (2023). Prevalence of adulteration in dietary supplements and recommendations for safe supplement practices in sport. Front. Sports Act. Living.

[17] Martinez-Sanz J.M., Sospedra I., Ortiz C.M., Baladia E., Gil-Izquierdo A., Ortiz-Moncada R. (2017). Intended or Unintended Doping? A Review of the Presence of Doping Substances in Dietary Supplements Used in Sports. Nutrients.

[18] Hei Y., Xie Y. (2025). Effects of exercise combined with different dietary interventions on cardiovascular health a systematic review and network meta-analysis. BMC Cardiovasc. Disord..

[19] Charkot J., Bieńkowski W., Kusy B., Bieńkowski M., Charkot M., Kucharczyk P., Jaszczuk I., Retman P. (2025). The impact of maximal oxygen uptake (VO_2_max) on athletic performance and health—A review. Qual. Sport.

[20] Marques-Vidal P., Tsampasian V., Cassidy A., Biondi-Zoccai G., Chrysohoou C., Koskinas K., Verschuren W.M.M., Czapla M., Kavousi M., Kouvari M. (2025). Diet and nutrition in cardiovascular disease prevention: A scientific statement of the European Association of Preventive Cardiology and the Association of Cardiovascular Nursing & Allied Professions of the European Society of Cardiology. Eur. J. Prev. Cardiol..

[21] Tu D., Song Z., Ren C., Hu Y., Jin Q., Wang Y. (2025). Joint association of antioxidant intakes from diet and supplements and sedentary behavior with all-cause and cardiovascular disease mortality among US adults. BMC Public Health.

[22] Borecki M. (2025). Legal and ethical analysis of dietary supplement use in the light of anti-doping regulations. Qual. Sport.

[23] Backhouse S.H. (2023). A Behaviourally Informed Approach to Reducing the Risk of Inadvertent Anti-doping Rule Violations from Supplement Use. Sports Med..

[24] Vasan R.S., van den Heuvel E. (2022). Differences in estimates for 10-year risk of cardiovascular disease in Black versus White individuals with identical risk factor profiles using pooled cohort equations: An in silico cohort study. Lancet Digit. Health.

[25] Jung E., Kong S.Y., Ro Y.S., Ryu H.H., Shin S.D. (2022). Serum Cholesterol Levels and Risk of Cardiovascular Death: A Systematic Review and a Dose-Response Meta-Analysis of Prospective Cohort Studies. Int. J. Env. Res. Public Health.

[26] Fang S.C., Wu Y.L., Tsai P.S. (2020). Heart Rate Variability and Risk of All-Cause Death and Cardiovascular Events in Patients with Cardiovascular Disease: A Meta-Analysis of Cohort Studies. Biol. Res. Nurs..

[27] Fatima F., Waheed S., Asifullah M.A., Hassan T., Samo N.H., Zamurd B. (2024). Nutritional Strategies for Optimizing Performance in Endurance Sports: A Case Study of Marathon Runners. Int. J. Biochem. Res. Rev..

[28] Ulrich R., Cleret L., Comstock R.D., Kanayama G., Simon P., Pope H.G. (2023). Assessing the Prevalence of Doping Among Elite Athletes: An Analysis of Results Generated by the Single Sample Count Method Versus the Unrelated Question Method. Sports Med. Open.

[29] Windfeld-Mathiasen J., Heerfordt I.M., Dalhoff K.P., Andersen J.T., Andersen M.A., Johansson K.S., Biering-Sorensen T., Olsen F.J., Horwitz H. (2025). Cardiovascular Disease in Anabolic Androgenic Steroid Users. Circulation.

[30] Borowiec A., Waluszewska I., Jurkiewicz M., Szczurek-Wasilewicz W. (2025). Impact of Anabolic–Androgenic Steroid Abuse on the Cardiovascular System: Molecular Mechanisms and Clinical Implications. Int. J. Mol. Sci..

[31] Iliakis P., Stamou E., Kasiakogias A., Manta E., Sakalidis A., Vakka A., Theofilis P., Kourti F.E., Konstantinidis D., Dimitriadis K. (2025). Anabolic–Androgenic Steroids Induced Cardiomyopathy: A Narrative Review of the Literature. Biomedicines.

[32] Te Hoonte F., Spronk M., Sun Q., Wu K., Fan S., Wang Z., Bots M.L., Van der Schouw Y.T., Uijl A., Vernooij R.W.M. (2024). Ideal cardiovascular health and cardiovascular-related events: A systematic review and meta-analysis. Eur. J. Prev. Cardiol..

[33] Ng C.Y.H., Tan B.Y.Q., Teo Y.N., Teo Y.H., Syn N.L.X., Leow A.S.T., Ho J.S.Y., Chan M.Y., Wong R.C.C., Chai P. (2022). Myocardial infarction, stroke and cardiovascular mortality among migraine patients: A systematic review and meta-analysis. J. Neurol..

[34] Pant A., Gribbin S., McIntyre D., Trivedi R., Marschner S., Laranjo L., Mamas M.A., Flood V., Chow C.K., Zaman S. (2023). Primary prevention of cardiovascular disease in women with a Mediterranean diet: Systematic review and meta-analysis. Heart.

[35] Hayes R.B., Lim C., Zhang Y., Cromar K., Shao Y., Reynolds H.R., Silverman D.T., Jones R.R., Park Y., Jerrett M. (2020). PM2.5 air pollution and cause-specific cardiovascular disease mortality. Int. J. Epidemiol..

[36] Di Fazio N., Volonnino G., Treglia M., Delogu G., Bubbico T., Rinaldi R., La Russa R., Maiese A. (2025). Forensic approach in cases of anabolic-androgenic steroid abuse and cardiovascular mortality: Insights from autopsy, histopathology, immunohistochemistry and toxicology. Front. Cardiovasc. Med..

[37] Vauhkonen P.K., Kiiskila J.M., Hytonen S., Koskela R., Mayranpaa M.I., Lindroos K.M. (2025). Microscopic cardiac pathology in forensic autopsies: A comparative study of anabolic androgenic steroid users and non-users. Int. J. Leg. Med..

[38] Morena D., Bonsignore A., Santurro A., Ventura F., Fineschi V. (2025). Sudden cardiac testosterone-related death involving a young bodybuilder: Autopsy, histopathological and toxicological findings. Egypt. J. Forensic Sci..

[39] Grassi S., Vaiano F., Dimitrova A., Vullo C., Croce E.B., Rossi R., Arena V., Strano Rossi S., Campuzano O., Brugada R. (2025). Fatal intoxications and inherited cardiac disorders in the young: Where to draw the line?. Int. J. Leg. Med..

[40] Sooltan I., Bulugahapitiya S. (2025). Devastating impact of performance-enhancing drugs: A case of severe heart failure in a young bodybuilder with body dysmorphic disorder. Br. J. Cardiol..

[41] Tarnovskaya S.I., Zhorov B.S. (2025). Predicting the Damaging Potential of Uncharacterized KCNQ1 and KCNE1 Variants. Int. J. Mol. Sci..

[42] Vanoye C.G., Desai R.R., Jairam N., Ghabra N., Meiler J., Sanders C.R., George A.L. (2022). Comprehensive functional evaluation of KCNE1 variants using automated patch clamp recording. Biophys. J..

[43] Greenberg S.M., Ziai W.C., Cordonnier C., Dowlatshahi D., Francis B., Goldstein J.N., Hemphill J.C., Johnson R., Keigher K.M., Mack W.J. (2022). 2022 Guideline for the Management of Patients with Spontaneous Intracerebral Hemorrhage: A Guideline from the American Heart Association/American Stroke Association. Stroke.

[44] Wang D., Jiang R., Kang K., Wang A., Zhang X., Lu J., Zhao X. (2023). Association of severity and prognosis with elevated blood pressure and heart rate levels in patients with intracerebral hemorrhage. BMC Neurol..

[45] Cricco R., Segreti A., Stirpe E., Ferro A., Ciancio M., Cipriani F., Fossati C., Ussia G.P., Pigozzi F., Grigioni F. (2025). Performance-Enhancing Effects of Inhaled Medications: Implications for Heart, Muscle Function, and Doping Detection in Athletes. J. Funct. Morphol. Kinesiol..

[46] Lago S., de Beukelaar T.T., Casetta I., Arcara G., Mantini D. (2025). Heart Rate Variability and Autonomic Dysfunction After Stroke: Prognostic Markers for Recovery. Biomedicines.

[47] Wang X., Chen L., Wei J., Zheng H., Zhou N., Xu X., Deng X., Liu T., Zou Y. (2025). The immune system in cardiovascular diseases: From basic mechanisms to therapeutic implications. Signal Transduct. Target. Ther..

[48] Maida C.D., Norrito R.L., Rizzica S., Mazzola M., Scarantino E.R., Tuttolomondo A. (2024). Molecular Pathogenesis of Ischemic and Hemorrhagic Strokes: Background and Therapeutic Approaches. Int. J. Mol. Sci..

[49] Boehme A.K., Esenwa C., Elkind M.S. (2017). Stroke Risk Factors, Genetics, and Prevention. Circ. Res..

[50] van Loon I.G., van der Schouw Y.T., Handoko M.L., Verschuren W.M.M., Uijl A. (2025). Comparing Life's Simple 7 and Life's Essential 8 with Risk of Heart Failure. JACC Adv..

[51] Medical N. ABI Testing in Athletes: When High Performers Have Hidden PAD. https://newman-medical.com/abi-in-athletes/.

[52] Fioretti V., Gerardi D., Giugliano G., Di Fazio A., Stabile E. (2023). Focus on Prevention: Peripheral Arterial Disease and the Central Role of the Cardiologist. J. Clin. Med..

[53] Tao M., Zhang Y., Li Q., Feng X., Ping C. (2024). Association of lipids and lipid-lowering drugs with peripheral arterial disease: A Mendelian randomization study. J. Clin. Lipidol..

[54] Aftab U., Duseja N. (2025). A Case of Advanced Peripheral Artery Disease with Advanced Coronary Artery Disease—A case report. Clin. Case Rep. Stud..

[55] Butler T., Twine C.P., Rodriguez-Mateos A., Heiss C. (2025). Feeding vascular health: Role of nutrition in peripheral artery disease prevention and management. Eur. J. Prev. Cardiol..

[56] Trommelen J., van Lieshout G.A.A., Nyakayiru J., Holwerda A.M., Smeets J.S.J., Hendriks F.K., van Kranenburg J.M.X., Zorenc A.H., Senden J.M., Goessens J.P.B. (2023). The anabolic response to protein ingestion during recovery from exercise has no upper limit in magnitude and duration in vivo in humans. Cell Rep. Med..

[57] Alzu'bi A., Almahasneh F., Khasawneh R., Abu-El-Rub E., Baker W.B., Al-Zoubi R.M. (2024). The synthetic cannabinoids menace: A review of health risks and toxicity. Eur. J. Med. Res..

[58] Abdelaal G.M.M., Shehata S.A., Ali S.M., Moawed D.M.N.A. (2025). Genetic variants associated with sudden cardiac death in the young: A systematic review. Egypt. J. Forensic Sci..

[59] Zhang W., Gan D., Huo S., Chen P. (2024). Unraveling the discrepancies between REDUCE-IT and STRENGTH trials with omega-3 fatty acids: New analytical approaches. Front. Nutr..

[60] Wu Y., Zhang L., Li J., Xue B., Shang W., Lu Y. (2025). Optimal calorie restriction threshold: Effect of FATmax exercise combined with different proportions of calorie restriction on hypercholesterolemia. Front. Physiol..

[61] Lyngbaek M.P.P., Legaard G.E., Nielsen N.S., Durrer C., Almdal T.P., Lund M.A.V., Liebetrau B., Ewertsen C., Lauridsen C., Solomon T.P.J. (2025). Effects of caloric restriction with different doses of exercise on fat loss in people living with type 2 diabetes: A secondary analysis of the DOSE-EX randomized clinical trial. J. Sport. Health Sci..

[62] Backhouse S.H. (2024). No Guarantees! Supporting Athletes to Reduce the Risk of Unintentional Doping from Supplement Use. Sport Sci. Exch..

[63] Maughan R.J., Burke L.M., Dvorak J., Larson-Meyer D.E., Peeling P., Phillips S.M., Rawson E.S., Walsh N.P., Garthe I., Geyer H. (2018). IOC consensus statement: Dietary supplements and the high-performance athlete. Br. J. Sports Med..

[64] Geyer H., Gmeiner G. (2019). Nutritional Supplements. Still a Risk for Inadvertent Doping.

[65] Goldstein E.R., Stout J.R., Wells A.J., Antonio J., Vasenina E., Fukuda D.H. (2023). Carbohydrate-Protein drink is effective for restoring endurance capacity in masters class athletes after a two-Hour recovery. J. Int. Soc. Sports Nutr..

[66] Tan A.Y., Hamzah S.H., Huang C.Y., Kuo C.H. (2021). Pre-exercise Carbohydrate Drink Adding Protein Improves Post-exercise Fatigue Recovery. Front. Physiol..

[67] Cao W., He Y., Fu R., Chen Y., Yu J., He Z. (2025). A Review of Carbohydrate Supplementation Approaches and Strategies for Optimizing Performance in Elite Long-Distance Endurance. Nutrients.

[68] Parish S., Mafham M., Offer A., Barton J., Wallendszus K., Stevens W., Buck G., Haynes R., Collins R., Bowman L. (2020). Effects of Omega-3 Fatty Acid Supplements on Arrhythmias. Circulation.

[69] Lazzarin T., Martins D., Ballarin R.S., Monte M.G., Minicucci M.F., Polegato B.F., Zornoff L. (2023). The Role of Omega-3 in Attenuating Cardiac Remodeling and Heart Failure through the Oxidative Stress and Inflammation Pathways. Antioxidants.

[70] Sawka M.N., Cheuvront S.N., Carter R. (2005). Human water needs. Nutr. Rev..

[71] Bentestuen M.S., Weis C.N., Jeppesen C.B., Thiele L.S., Thirstrup J.P., Cordero-Solorzano J., Jensen H.K., Starnawska A., Hauser A.S., Gasse C. (2025). Pharmacogenomic markers associated with drug-induced QT prolongation: A systematic review. Pharmacogenomics.

[72] Mansoor S.I.U. (2025). Balancing the benefits and risks of traditional medicine in sports: A study of anti-doping controls and compliance. Int. Sports Law. J..

[73] Varillas-Delgado D., Del Coso J., Gutierrez-Hellin J., Aguilar-Navarro M., Munoz A., Maestro A., Morencos E. (2022). Genetics and sports performance: The present and future in the identification of talent for sports based on DNA testing. Eur. J. Appl. Physiol..

[74] Cao Y., Sun J., Wang X., Zhang X., Tian H., Huang L., Huang Z., Zhang Y., Zhang J., Li L. (2024). The double-edged nature of nicotine: Toxicities and therapeutic potentials. Front. Pharmacol..

[75] Collet R., van Grootel J., van Dongen J., Wiertsema S., Ostelo R., van der Schaaf M., Lazzari E., Geleijn E., Major M., van der Leeden M. (2025). The Impact of Multidisciplinary Transitional Care Interventions for Complex Care Needs: A Systematic Review and Meta-Analysis. Gerontologist.

[76] Esposito M., Licciardello G., Privitera F., Iannuzzi S., Liberto A., Sessa F., Salerno M. (2021). Forensic Post-Mortem Investigation in AAS Abusers: Investigative Diagnostic Protocol. A Systematic Review. Diagnostics.

[77] Hernandez-Guerra A.I., Tapia J., Menendez-Quintanal L.M., Lucena J.S. (2019). Sudden cardiac death in anabolic androgenic steroids abuse: Case report and literature review. Forensic Sci. Res..

[78] Torrisi M., Pennisi G., Russo I., Amico F., Esposito M., Liberto A., Cocimano G., Salerno M., Li Rosi G., Di Nunno N. (2020). Sudden Cardiac Death in Anabolic-Androgenic Steroid Users: A Literature Review. Medicina.

[79] Li M.Y., Peng L.M., Chen X.P. (2022). Pharmacogenomics in drug-induced cardiotoxicity: Current status and the future. Front. Cardiovasc. Med..

[80] Casparsen C. (2024). The role of toxicology in forensic investigations. Int. J. Forensic Med..

[81] Langman L.J., Snozek C.L.H. (2012). LC-MS in Drug Analysis: Methods and Protocols.

[82] AlDossary M., AlShamsi G., AlFares M., Alakhras H., Alsahab A., Alshaikhi Y., Alamari A., Issa S.Y. (2025). Postmortem forensic toxicology: A retrospective investigation from Dammam-Saudi Arabia. Egypt. J. Forensic Sci..

[83] Crisan D., Avram L., Morariu-Barb A., Grapa C., Hiriscau I., Craciun R., Donca V., Nemes A. (2025). Sarcopenia in MASLD-Eat to Beat Steatosis, Move to Prove Strength. Nutrients.

[84] Truver M.T., Chronister C.W., Davis G.G., Gray T.R., Hartman R.L., Kahl J.H., Karschner E.L., Kerrigan S., Kronstrand R., Krotulski A.J. (2025). Application of professional best practices in postmortem forensic toxicology. J. Anal. Toxicol..

[85] Avram M., Moran J.H., Marin S.J., Tackett B. (2025). Drug Screening and Confirmation Using Two LC Columns and Identical LC-MS/MS Conditions Combined into a Single Streamlined Forensic Workflow.

[86] Martinez-Matilla M., Blanco-Verea A., Torres M., Ramos-Luis E., Gil R., Bermejo A., Hirata M.H., Brisighelli F., Paramo M., Carracedo A. (2017). P5857Study of pharmacodynamic and pharmacokinetic-mediated genetic susceptibility to drug-induced arrhythmia and sudden cardiac death. Eur. Heart J..

[87] Di Nunno N., Esposito M., Argo A., Salerno M., Sessa F. (2021). Pharmacogenetics and Forensic Toxicology: A New Step towards a Multidisciplinary Approach. Toxics.

[88] Ingelman-Sundberg M., Nebert D.W., Lauschke V.M. (2023). Emerging trends in pharmacogenomics: From common variant associations toward comprehensive genomic profiling. Hum. Genom..

[89] Battal D., Yukseloglu E.H., Alkas F.B., Firat S.S. (2022). Pharmacogenomics perspective for forensic toxicology: A mini review. Med. Sci..

[90] Padigepati S.R., Stafford D.A., Tan C.A., Silvis M.R., Jamieson K., Keyser A., Nunez P.A.C., Nicoludis J.M., Manders T., Fresard L. (2024). Scalable approaches for generating, validating and incorporating data from high-throughput functional assays to improve clinical variant classification. Hum. Genet..

[91] Ma K., Gauthier L.O., Cheung F., Huang S., Lek M. (2024). High-throughput assays to assess variant effects on disease. Dis. Model. Mech..

[92] Baracaldo-Santamaria D., Llinas-Caballero K., Corso-Ramirez J.M., Restrepo C.M., Dominguez-Dominguez C.A., Fonseca-Mendoza D.J., Calderon-Ospina C.A. (2021). Genetic and Molecular Aspects of Drug-Induced QT Interval Prolongation. Int. J. Mol. Sci..

[93] Richards-Brown M., Wei Y., Abidoph R., Varney L., Cotic M., Murtough S., Panconesi D., Mills D., Richards-Belle A., Saadullah Khani N. (2025). Patient and clinician perspectives on pharmacogenetic testing for antipsychotics. Front. Pharmacol..

[94] Vlahovich N., Hughes D.C., Griffiths L.R., Wang G., Pitsiladis Y.P., Pigozzi F., Bachl N., Eynon N. (2017). Genetic testing for exercise prescription and injury prevention: AIS-Athlome consortium-FIMS joint statement. BMC Genom..

[95] Mathews N.M. (2018). Prohibited Contaminants in Dietary Supplements. Sports Health.

